# Life-long tailoring of diagnosis and management of patients with idiopathic ventricular fibrillation—future perspectives in research

**DOI:** 10.1007/s12471-018-1123-3

**Published:** 2018-06-07

**Authors:** L. J. Blom, P. G. A. Volders, A. A. Wilde, R. J. Hassink

**Affiliations:** 1 Department of Cardiology, University Medical Center, Utrecht, The Netherlands; 2 Department of Cardiology, Cardiovascular Research Institute Maastricht (CARIM), Maastricht University Medical Center, Maastricht, The Netherlands; 3 Department of Clinical and Experimental Cardiology, Heart Centre, Academic Medical Center (AMC), Amsterdam, The Netherlands

**Keywords:** Ventricular fibrillation, Cardiac electrophysiology, Sudden cardiac arrest, Cardiovascular diagnostic techniques, Early diagnosis, Genetic testing

## Abstract

The diagnosis and management of idiopathic ventricular fibrillation is challenging, as it requires extensive diagnostic testing and offers few curative options due to unknown underlying disease. The resulting population is a heterogeneous group of patients with a largely unknown natural history. Structural patient characterisation, follow-up and innovations in diagnostic testing can improve our understanding of the disease mechanisms of idiopathic ventricular fibrillation, detect underlying disease during follow-up and aid in therapeutic management. Recently, initiatives have been launched in the Netherlands to investigate the role of high-resolution non-invasive electrocardiographic imaging and genetic and familial screening in idiopathic ventricular fibrillation.

## Introduction

Idiopathic ventricular fibrillation is a rare cause of sudden cardiac arrest, defined as ‘resuscitated cardiac arrest, preferably with documentation of ventricular fibrillation, for which known cardiac, respiratory, metabolic, and toxicological aetiologies have been excluded through clinical evaluation’ [1].

In general, patients with idiopathic ventricular fibrillation are young (average age: 38) and present with a structurally normal heart. Specific genetic disorders and diseases, such as primary arrhythmia syndromes, should be evaluated in these patients [1–3].

Limited data are available on the natural history of idiopathic ventricular fibrillation, including identification of putative pathogenic mutations, and management of patients with idiopathic ventricular fibrillation and their family members [2, 4].

Idiopathic ventricular fibrillation is a *diagnosis of exclusion* and therefore patients undergo a broad range of diagnostic tests. We proposed a flowchart to standardise diagnostic testing in patients with idiopathic ventricular fibrillation [2]. Routine testing comprises electrocardiogram (ECG), blood chemistry (cardiac enzymes, electrolytes, and thyroid function), toxicology screening, chest X‑ray, echocardiography, exercise testing, Holter or telemetry monitoring, coronary angiography with or without ventriculography, and magnetic resonance imaging. When these tests reveal no abnormalities, provocation tests for Brugada syndrome (ajmaline/flecainide test) and coronary artery spasm (ergonovine/acetylcholine test) are recommended. Additional testing, such as endomyocardial biopsy, electrophysiological studies and genetic testing, is under debate, as their diagnostic value in idiopathic ventricular fibrillation is uncertain [2].

The yield of genetic testing in idiopathic ventricular fibrillation is interesting, as it contributed immensely to the detection of primary arrhythmia syndromes. However, a clinical suspicion, based on phenotype, should guide genetic testing. Even with next generation sequencing, which screens large gene panels at once, the yield is minimal and variants of uncertain significance are often detected [5]. Proposed genetic testing entails a basic panel of SCN5A, the most common long QT genes (KCNQ1 and KCNH2), RyR2, and CALM1 in patients with exercise-induced ventricular fibrillation. In patients with a negative phenotype, we recommend screening of SCN5A, KCNQ1, and KCNH2 [2]. In the central part of the Netherlands and in families originating from the Gouda region, DPP6 screening is recommended as the DPP6 haplotype accounts for a large part of the idiopathic ventricular fibrillation population (over 25%) in this area [6].

In current practice, patients are diagnosed with idiopathic ventricular fibrillation after limited diagnostic testing [7, 8]. Therefore, follow-up and re-evaluation of the diagnosis are important aspects of the management of patients with idiopathic ventricular fibrillation. Of patients with idiopathic ventricular fibrillation, 7 to 35% reveal a different diagnosis during follow-up due to disease progression or more extensive or more sophisticated diagnostic evaluation [9–13]. Since the underlying disease substrate is often not known, discovery of new disease entities and novel diagnostic techniques can reveal causes not detectable or known during initial evaluation.

## A patient initially diagnosed with idiopathic ventricular fibrillation

**Fig. 1:**
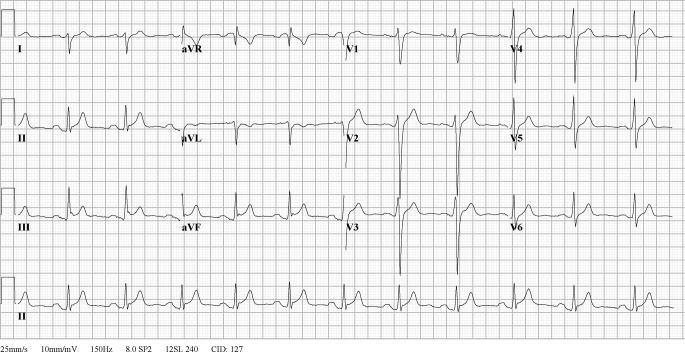
12-lead electrocardiogram while off anti-arrhythmic drugs, showing normal sinus rhythm, QRS right axis deviation, QRS width 140 ms and prolonged terminal activation duration in V2 (70 ms). Clear J‑point and ST elevation in II, III, and aVF

To support the importance of life-long tailoring of diagnosis and management of patients with idiopathic ventricular fibrillation we describe a case of idiopathic ventricular fibrillation presented to our centre:

A 24-year-old man collapsed twice in one year while playing a football match. The first time, in 1991, he regained consciousness shortly after resuscitation was started, and clinical evaluation was inconclusive. The second time, in 1992, he received 2 external defibrillator shocks because of ventricular fibrillation. After successful resuscitation he was admitted for diagnostic evaluation. Electrocardiography showed a sinus rhythm with intraventricular conduction delay (QRS 140 ms), including prolonged terminal activation duration (TAD 70 ms), and J‑point elevation in the inferior leads (Fig. 1). History, Holter monitoring and exercise test were unremarkable, no ventricular extrasystoles were reported. Family history was negative for sudden cardiac death. Echocardiogram showed normal cardiac function and normal right and left ventricular dimensions, with a local abnormality under the tricuspid valve. Transoesophageal echocardiogram characterised this as prolapse of a valve leaflet. During electrophysiologic study, a prolonged HV interval of 60–70 ms was measured. Right ventricular stimulation induced polymorphic ventricular tachycardia starting as monomorphic ventricular tachycardia with left bundle branch block morphology and superior axis. Other diagnostic tests, including coronary angiogram, myocardial biopsy and ergonovine provocation test, were normal. He received an implantable cardioverter-defibrillator (ICD) and was discharged. In the absence of an obvious aetiology, he was diagnosed with idiopathic ventricular fibrillation.

**Fig. 2:**
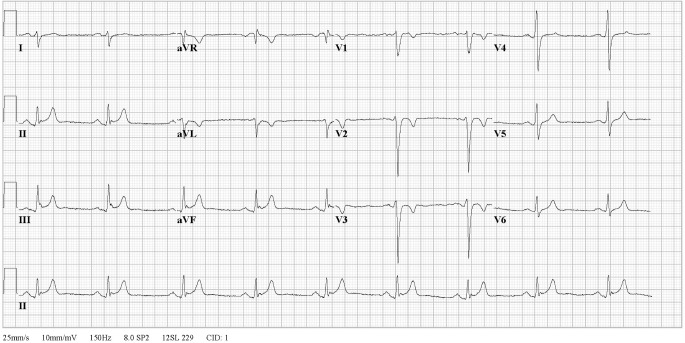
12-lead electrocardiogram while off anti-arrhythmic drugs after 10 years of follow-up, showing negative T waves in V1–V3. Terminal activation delay and J‑point elevation are still present

**Fig. 3:**
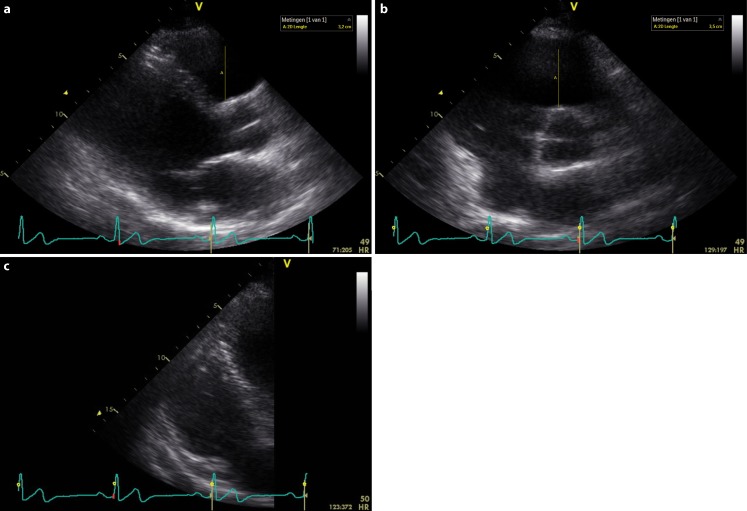
Echocardiogram in 2008 showing dilatation of the right ventricle and right ventricular outflow tract. **a** PLAX RVOT, **b** PSAX RVOT, **c** AP4CH-focused RV. *PLAX* parasternal long axis view*, RVOT* right ventricular outflow tract,* PSAX* parasternal short axis view*, AP4CH* apical 4 chamber view,* RV* right ventricle

During twenty-five years of follow-up, he remained free of ventricular tachyarrhythmias. Ten years after the initial event negative T waves were recorded in V1–V3 (Fig. 2). On follow-up echocardiogram, there were no longer signs of tricuspid valve prolapse, but careful re-evaluation of the imaging showed subtricuspid dyskinesia. Sixteen years after the event right ventricular dilatation was found (echocardiogram 2008: dilated right ventricle (RV), regional hypokinesia of the right ventricular outflow tract (RVOT) and anterior right ventricle, parasternal long axis view RVOT: 32 mm, parasternal short axis view RVOT: 35 mm, Fig. 3). Consecutive targeted molecular genetic testing revealed no pathogenic mutations in desmosomal genes, but the sodium channel gene SCN5A revealed a p.Leu729del mutation. Familial co-segregation supported pathogenicity of this mutation [14].

After re-evaluation of the diagnostic workup he was diagnosed with arrhythmogenic cardiomyopathy according to the 2010 International Task Force Criteria [15]: T‑wave inversion in right precordial leads (1 major), ventricular tachycardia with left bundle branch block configuration and superior axis (1 major), structural abnormalities on echocardiogram (1 major) and prolonged terminal activation duration (1 minor).

As this case shows, the diagnosis of underlying disease in idiopathic ventricular fibrillation is a dynamic process in which the initial evaluation has not led to a satisfactory result and over time underlying disease can become manifest. During long-term follow-up, conventional diagnostic tests can detect progression of underlying disease, new diagnostic tests can aid in detecting underlying disease and increased understanding of the disease can lead to diagnosis and additional management options.

## Future perspectives in idiopathic ventricular fibrillation research

### The role of a national/international database

The current population with idiopathic ventricular fibrillation is a rare, heterogeneous group, where diagnostic certainty depends on the extent of workup to exclude other underlying causes. The prevalence of idiopathic ventricular fibrillation in the Netherlands is unknown, but it is estimated at 1,000 patients nationwide. At this moment, little is known about the diagnostic certainty and extent of diagnostic workup of patients with idiopathic ventricular fibrillation in the Netherlands. A comprehensive registry of patients with idiopathic ventricular fibrillation with national and international coverage should be established to study the prevalence and characteristics of these patients. The database should include information on performed diagnostic tests to assess the reliability of the diagnosis and clues for underlying disease.

Through advances in the understanding of primary electrical syndromes and the discovery of novel founder mutations in idiopathic ventricular fibrillation, subgroups with a specific underlying disease can be identified and diagnosed and managed appropriately, improving prognosis and preventing recurrent events.

### Design of the national consortium study VIGILANCE under the Netherlands Cardiovascular Research Initiative

**Fig. 4:**
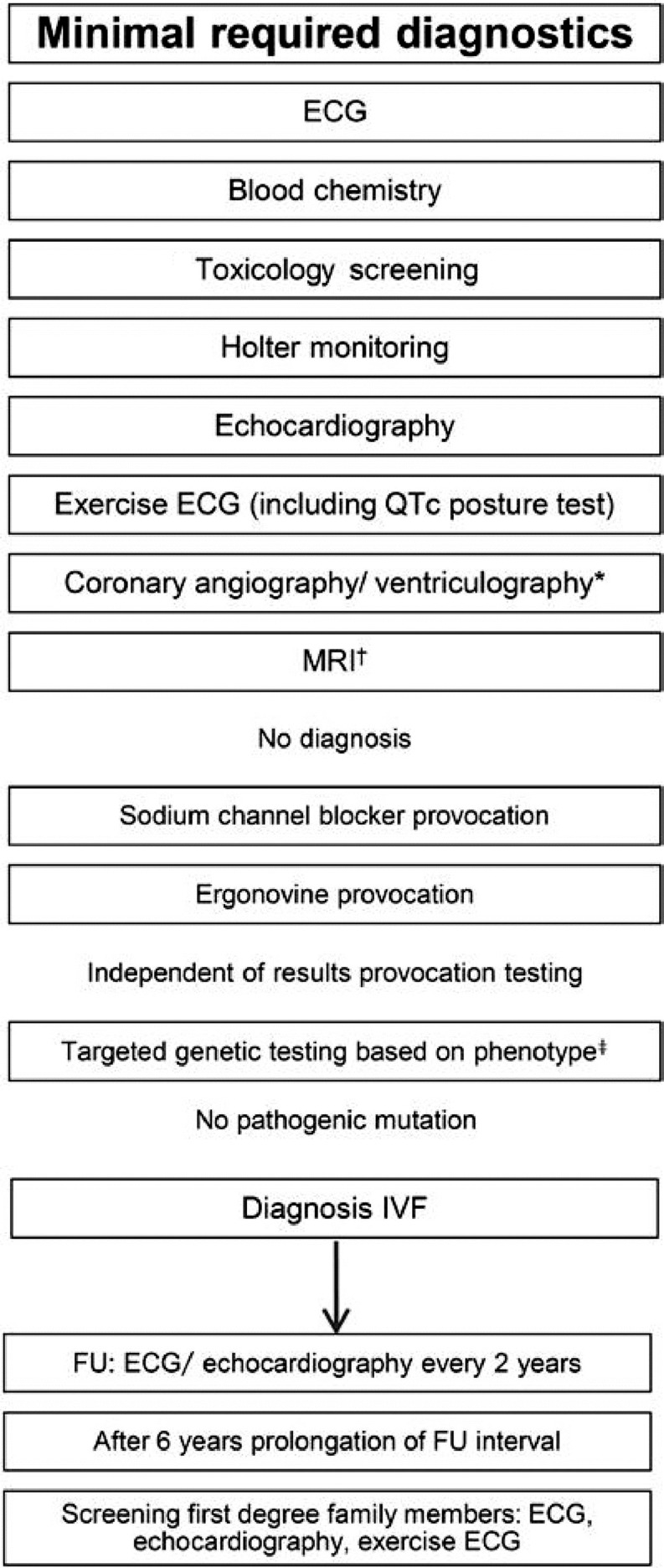
Flowchart for the diagnosis and follow-up of patients with idiopathic ventricular fibrillation. *In young patients (<45 years) without risk factors for coronary artery disease, coronary computed tomography (CT) angiography is an alternative diagnostic tool to exclude coronary artery disease. *ECG* electrocardiogram,* IVF* idiopathic ventricular fibrillation,* MRI* magnetic resonance imaging*, FU* follow-up (from Visser et al. [2])

The VIGILANCE (*non-in*v*as*i*ve electrocardio*g*raph*i*c imaging for individua*l*s *a*t risk for appare*n*tly idiopathi*c* v*e*ntricular fibrillation*) project focuses on establishing a Dutch national registry of patients with idiopathic ventricular fibrillation. The registry’s foundation has been laid and already includes 8 university medical centres and tertiary centres. Our aim is to include all Heart Centres of the University Medical Centres and all ICD implant centres in the Netherlands. We will visit these institutes and collect patients from existing electronic patient records. In the beginning of the inclusion process, survivors of cardiac arrest due to ventricular fibrillation in whom at least 50% of routine diagnostic tests have been performed and no underlying disease has been found will be included in the registry. These survivors will be screened using the diagnostic flowchart published by our group (Fig. 4) and comprehensive data review will be performed to assess the completeness of diagnostic testing. Next, we will discuss the missing diagnostics with the treating physician and finalise the diagnostic process, excluding patients with alternative diagnoses. From this cohort of patients with clear idiopathic ventricular fibrillation with complete diagnostic workup, we will collect data on long-term follow-up of patients with idiopathic ventricular fibrillation, including ICD therapy and clinical outcome. Furthermore, this registry will be a starting point for future research on the arrhythmogenesis of idiopathic ventricular fibrillation.

### Follow-up of patients with idiopathic ventricular fibrillation

During follow-up, patients will be monitored continuously and undergo diagnostic tests at regular intervals, which will provide valuable insight into natural disease course, specific subgroup identification and disease severity. Data on the appropriate burden of ICD shock and ICD-related complications can be used to assess the need and benefit of ICD implantation.

### Genetics and family screening

Arrhythmic risk assessment in asymptomatic patients is difficult, especially with unknown underlying disease. However, many patients with idiopathic ventricular fibrillation show a familial risk for sudden cardiac death and there are several well-characterised populations (e. g. *DPP6, SCN5A*) in the Netherlands [6, 16]. Data from genetic testing in patients with idiopathic ventricular fibrillation and their family members will be used to assess the yield of genetic testing, the detection of variants of uncertain clinical significance and their contribution to arrhythmic risk in this population.

### Body surface mapping

Absence of known arrhythmic substrates in patients with idiopathic ventricular fibrillation provides an opportunity for novel, preferably non-invasive, techniques. Electrocardiographic imaging (ECGI) is an emerging high-resolution non-invasive imaging modality for mapping the electrical activity in the heart [17, 18]. ECGI employs mathematical formulations to reconstruct the electrical activity at the level of the heart muscle, from extensive body-surface electrocardiograms and a digital, patient-specific body and heart geometry. It uses a model of the propagation of the electromagnetic field (from the heart to the body surface) to reconstruct the electrical source of the recorded body-surface ECGs [19].

In healthy individuals, ECGI demonstrated that dispersion is small, for example for repolarisation in humans [20, 21]. However, ECGI did show increased dispersion in several arrhythmogenic diseases. In long QT syndrome [20] and Brugada syndrome [21], patients had abnormally steep repolarisation gradients. Repolarisation gradients were steeper in symptomatic patients, suggesting increased dispersion is predictive for arrhythmia risk [20]. We have validated ECGI’s ability to assess depolarisation and repolarisation accurately. These results suggest ECGI can play a pivotal role in characterising arrhythmia mechanisms in patients with idiopathic ventricular fibrillation, leading to diagnosis and improved treatment. Moreover, it seems to have the potential to detect arrhythmogenic substrate in individuals before their first event, offering the possibility to diagnose and treat patients before sudden cardiac arrest occurs. This is especially relevant in family members of victims of sudden cardiac death who might have a predisposition for idiopathic ventricular fibrillation. Within the VIGILANCE project, we will screen patients with idiopathic ventricular fibrillation for arrhythmogenic substrate using ECGI and help identify family members at risk for arrhythmic events.

## References

[1] Priori SG, Wilde AA, Horie M (2013). HRS/EHRA/APHRS Expert consensus statement on the diagnosis and management of patients with inherited primary arrhythmia syndromes. Heart Rhythm.

[2] Visser M, van der Heijden JF, Doevendans PA (2016). Idiopathic ventricular fibrillation: The struggle for definition, diagnosis and follow-up. Circ. Arrhythmia Electrophysiol.

[3] Krahn AD, Healey JS, Chauhan V (2009). Systematic assessment of patients with unexplained cardiac arrest: Cardiac Arrest Survivors With Preserved Ejection Fraction Registry (CASPER). Circulation.

[4] Honarbakhsh S, Srinivasan N, Kirkby C (2016). Medium-term outcomes of idiopathic ventricular fibrillation survivors and family screening: a multicentre experience. Europace.

[5] Visser M, Dooijes D, van der Smagt JJ (2017). Next-generation sequencing of a large gene panel in patients initially diagnosed with idiopathic ventricular fibrillation. Heart Rhythm.

[6] ten Sande JN, Postema PG, Boekholdt SM (2016). Detailed characterization of familial idiopathic ventricular fibrillation linked to the DPP6 locus. Heart Rhythm.

[7] Champagne J, Geelen P, Philippon F, Brugada P (2005). Recurrent cardiac events in patients with idiopathic ventricular fibrillation, excluding patients with the Brugada syndrome. Bmc Med.

[8] Remme CA, Wever EFD, Wilde AAM (2001). Diagnosis and long-term follow-up of the Brugada syndrome in patients with idiopathic ventricular fibrillation. Eur Heart J.

[9] Herman ARM, Cheung C, Gerull B (2016). Outcome of apparently unexplained cardiac arrest: Results from investigation and follow-up of the prospective Cardiac Arrest Survivors With Preserved Ejection Fraction Registry. Circ Arrhythm Electrophysiol.

[10] Matassini VM, Krahn AD, Gardner M (2014). Evolution of clinical diagnosis in patients presenting with unexplained cardiac arrest or syncope due to polymorphic ventricular tachycardia. Heart Rhythm.

[11] Conte G, Caputo ML, Regoli F (2017). True idiopathic ventricular fibrillation in out-of-hospital cardiac arrest survivors in the Swiss Canton Ticino: prevalence, clinical features, and long-term follow-up. Europace.

[12] Visser M, van der Heijden JF, van der Smagt JJ (2016). Long-term outcome of patients initially diagnosed with idiopathic ventricular fibrillation. Circ. Arrhythmia Electrophysiol.

[13] Mewis C, Kuhlkamp V, Spyridopoulos I (1998). Late outcome of survivors of idiopathic ventricular fibrillation. Am J Cardiol.

[14] te Riele ASJM, Agullo-Pascual E, James CA (2017). Multilevel analyses of SCN5A mutations in arrhythmogenic right ventricular dysplasia/cardiomyopathy suggest non-canonical mechanisms for disease pathogenesis. Cardiovasc Res.

[15] Marcus FI, McKenna WJ, Sherrill D (2010). Diagnosis of arrhythmogenic right ventricular cardiomyopathy/dysplasia: Proposed modification of the task force criteria. Circulation.

[16] ter Bekke RMA, Isaacs A, Barysenka A (2017). Heritability in a SCN5A-mutation founder population with increased female susceptibility to non-nocturnal ventricular tachyarrhythmia and sudden cardiac death. Heart Rhythm.

[17] Ramanathan C, Ghanem RN, Jia P (2004). Noninvasive electrocardiographic imaging for cardiac electrophysiology and arrhythmia. Nat Med.

[18] Cluitmans MJM, Peeters RLM, Westra RL, Volders PGA (2015). Noninvasive reconstruction of cardiac electrical activity: Update on current methods, applications and challenges. Neth Heart J.

[19] Cluitmans MJM, Clerx M, Vandersickel N (2017). Physiology-based regularization of the electrocardiographic inverse problem. Med Biol Eng Comput.

[20] Vijayakumar R, Silva JNA, Desouza KA (2014). Electrophysiologic Substrate in Congenital Long QT Syndrome: Noninvasive Mapping With Electrocardiographic Imaging (ECGI). Circulation..

[21] Zhang J, Sacher F, Hoffmayer K (2015). Cardiac Electrophysiological Substrate Underlying the ECG Phenotype and Electrogram Abnormalities in Brugada Syndrome Patients. Circulation.

